# Integration of groundwater vulnerability with contaminants transport modeling in unsaturated zone, case study El-Sharqia, Egypt

**DOI:** 10.1007/s10661-023-11298-3

**Published:** 2023-05-25

**Authors:** Abdel-Hameed El-Aassar, Kamilia Hagagg, Rasha Hussien, Selda Oterkus, Erkan Oterkus

**Affiliations:** 1 Egypt Desalination Research Center of Excellence (EDRC) and Hydrogeochemistry Department, Desert Research Centre, Cairo, Egypt; 2 Egyptian Atomic Energy Authority (EAEA), Cairo, Egypt; 3 PeriDynamics Research Centre (PDRC), University of Strathclyde, Glasgow, UK

**Keywords:** Groundwater contamination hazard, Trace elements, Pollution, Unsaturated zone modeling, HYDRUS-1D

## Abstract

Nowadays, irrigation uses large amount of marginal wastewater due to continuous decline in fresh water supply. As a consequence, using this wastewater for different purposes can cause some adverse environmental impacts. Anthropogenic activities such as septic tanks, sewage ponds, and polluted drains have large influence on deterioration of shallow groundwater aquifers. So, construction of many wastewater treatment plants in these areas is mandatory to control and mitigate this deterioration. Groundwater vulnerability assessment maps and contamination simulation in unsaturated zone can be beneficial in understanding contaminants pathways and groundwater quality evolution. This work is mainly focused on aquifer vulnerability assessment to pollution and the role of vadose zone in attenuation of contaminants transport through it prior to groundwater seepage. Therefore, about 56 drainage and groundwater samples were collected and analyzed for potentially toxic elements. The most vulnerable sector was determined using GOD method revealing that central parts of the study area are the most threatened zones with some scattered sporadic zone of sensitivity to pollution and this was verified through the zonation of Pb, Fe, and Mn spatial concentrations. The leakage of these elements through the unsaturated zone was further simulated using HYDRUS-1D model for the next 10-year period to determine the extent of the pollution plumes and maximum concentration of these elements that percolate to the groundwater directly. The concentration of Fe, Pb, and Mn at the end of the simulation reached low concentrations at the bottom layer of the unsaturated zone.

## Introduction

Groundwater is globally a vital source for sustaining ecosystems to meet their water demands. However, its sustainability is being threatened by several factors such as mismanagement of overexploitation activities to cope with increasing developmental activities (UNESCO, 2018). This situation is very critical specifically in the region of Northern Africa which includes a total of five countries, Egypt, Algeria, Morocco, Tunisia, and Libya, in which humans’ activities depend essentially on water availability (World Resources Institute, 2015; UN, 2019; Belhassan, 2022).

Nearly 2.3 billion people are suffering from adequate sanitation and lack of safe drinking water. According to UNESCO (2018), nearly half of the developing countries population is exposed to polluted water sources that might threaten human health. In addition to this, about 80% of wastewater is released into the environment without adequate treatment (WHO, 2017; UNESCO, 2018).

Wastewater disposal facilities, dumpsites, and sewage ponds cause constant hazards that threaten the quality of groundwater especially in developing African countries. They have an adverse impact on environmental, health, and social development as they may affect the ecosystem’s balance and pose a risk to human health. The consequence is serious degradation of environmental components. Therefore, groundwater pollution protection is a primary goal at national and international scale.

In arid and semi-arid countries like Egypt, shallow groundwater aquifers are main water resources for human demands in many regions around Nile delta area. On the other hand, seepage from wastewater disposal systems, sewage ponds, and other anthropogenic activities are challenging problematic issues in this region. More attention is directed toward El-Sharkia in the northeast area of Cairo to raise its resident’s living standards. Recently, several water treatment and desalination plants have been constructed to supply this area with safe fresh water to expand urbanization, agriculture, and industrial scale. Encouraging such activities increases the pollution load at this area and might result in groundwater deterioration.

The unsaturated zone that is considered a protective layer above the saturated zone might hinder the contaminants dispersion to groundwater, as the amount of infiltrated water is highly controlled by soil physical properties. The term of groundwater vulnerability has started to be important in groundwater pollution problems science 1980 (Attia, 1985; Foster, 1987). It is defined as the natural ground characteristics (geological, hydrological, and hydrogeological characteristics) that determine capacity at which a contaminant introduced at the ground surface reaches and diffuses in groundwater (Vrba & Zaporozec, 1994). Groundwater vulnerability is classified into intrinsic or specific vulnerability (NRC, 1993). DRASTIC (Attia et al., 1985) and GOD (Foster, 1987) are the most popular parametric methods used in this field of study in which physical attributes (geological, hydrological, and hydrogeological characteristics) are assigned numerical scores or rating scores to build parameter or base geospatial maps. An overlay process to develop a vulnerability map with a range of vulnerability categories is based on the combination of these parameter maps.

From this context, groundwater vulnerability assessment maps and contamination simulation in unsaturated zone can be beneficial as an early alarming tool that can help in understanding contaminants pathways and groundwater quality evolution. They give more insights toward understanding and predicting the groundwater resources vulnerability to pollution, as well as the contaminants transport through vadose zone and their loads to groundwater system in the study area. This information is quite important for land-use planners and water decision makers to take actions regarding the protection of aquifers against contamination. Many studies have been carried out to investigate solute transport processes in the saturated zone such as Fitch and Jia (1996), Chiogna et al. (2010), Rolle et al. (2010), Gai et al. (2011), Kumahor et al. (2015), Hussien et al. (2017), and Zhuang et al. (2021).

As a result of continuous decline in the available fresh water for irrigation sector; marginal waters can be used for irrigation or any other industrial activity such as raising fish. The effect of using such kind of low-quality water in different purposes is rather dangerous on the environmental component as a whole. Studying and assessing the effect of drainage and sewage drains on the groundwater are crucial for the present and future management and planning of groundwater resources in the area. Several sporadic studies in different sectors were done on the study area. The recent research includes hydrochemical and groundwater quality evaluation (Embaby et al., 2014; Mansour, 2020), seasonal variation in microbiological and physiochemical characteristics of municipal wastewater (Mahgoub et al., 2015), soil productivity potentials (Rashed, 2016), environmental threats of an on-site sewage disposal on groundwater at Minia Al-Qamh (Atwa et al., 2015), sedimentological, hydrochemical studies of groundwater aquifer in El Salhyia area (Mabrouk et al., 2016), and hydrogeological investigations of the Quaternary aquifer (El-Sayed et al., 2011).

This work is primarily devoted and focused on assessing the pollution-related aquifer vulnerability, as well as the movement of contaminates through the vadose zone and predicting their impacts on groundwater quality for mitigation and controlling pollution seepage from wastewater disposal systems and other anthropogenic activities. This can be achieved through utilization of based GIS (Geographic Information System)/groundwater vulnerability indicator (GOD) to contamination for demarcation of most vulnerable zones in El-Sharqia in the northeast area of Cairo, and studying the contaminants behavior and transport in the unsaturated zone using HYDRUS 1D as unsaturated zone modeling tool.

## Site description

**Fig. 1:**
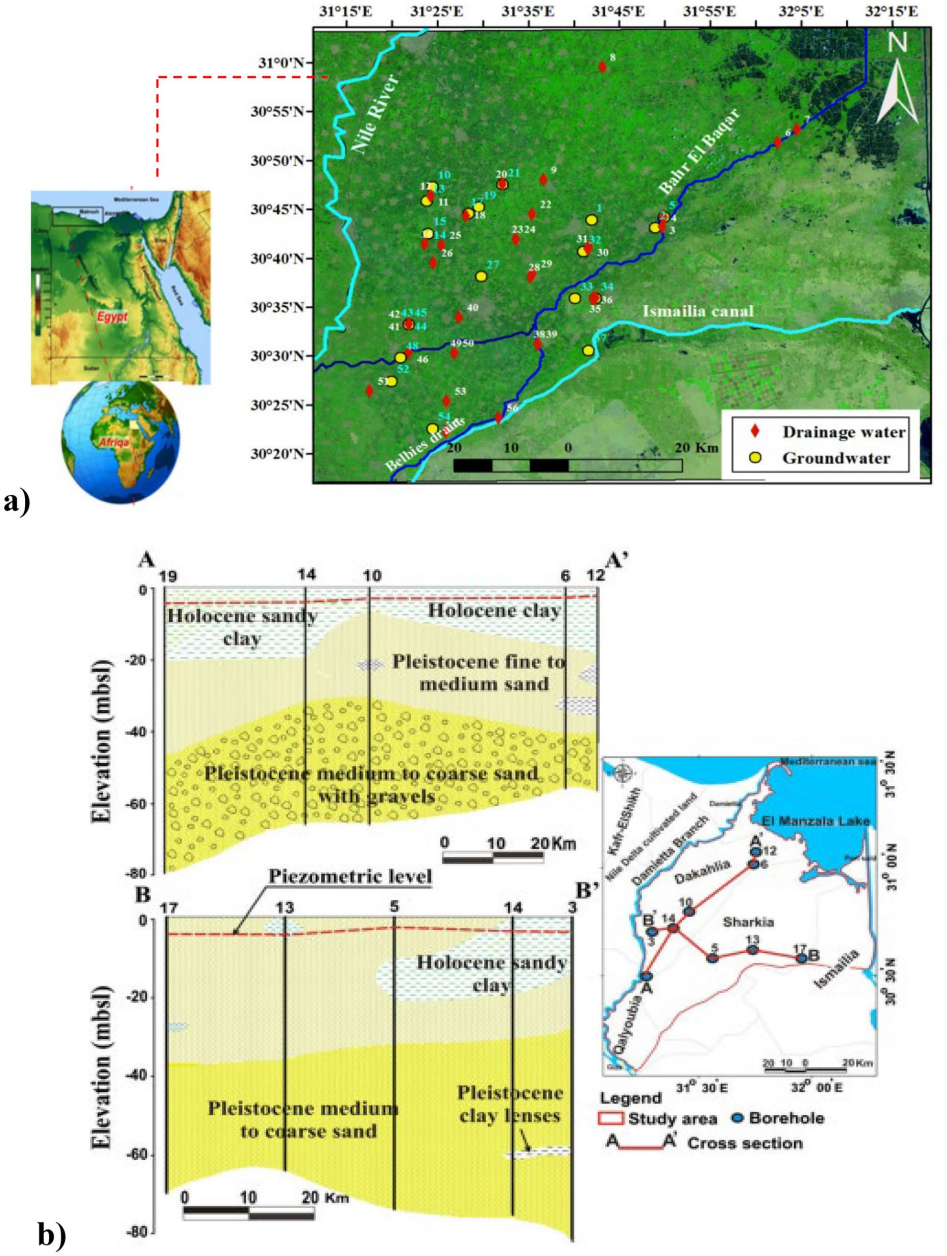
**a** Drainage and groundwater samples location map of the study area; **b** hydrogeological cross-sections describing the lateral litho-facies change of the Quaternary aquifer (after Elewa et al., 2012).

The study area (El Sharqia Governorate) is located at northeastern Nile delta of Egypt, approximately between longitudes 31° 15′ and 32° 15′ E and latitudes 30° 20′ and 31° N (Fig. 1a) covering 4911 Km^2^. Water treatment facilities with a capacity of 365 M.m^3^/year only provide water to 92.6% of its population. The average water consumption is 150/capita/day which is lower than Egypt’s average daily water consumption (220/capita/day) (IWA, 2014; CAPMATH, 2017). Rainfall is almost scarce and the fresh Nile water in irrigation canals is insufficient for irrigation activities. Treated wastewaters from agricultural and sewage drains are frequently applied for irrigation in the study area. The Holocene aquitard and the Pleistocene aquifer are considered the main aquifers in the study area. Pleistocene aquifer system is composed of sand and gravel with clay lenses which is capped by a semi-permeable Holocene aquitard and rests on an impermeable Pliocene aquiclude. The Quaternary aquifer is confined in the study area except at Rolling Plains. Its thickness varies from 300 m in the south to 600 m in the north and northwest. Seepage from canals and subsurface drainage are considered the major recharge sources. The groundwater level ranges from 8 to 3 m (above sea level) in the study area. The flow of groundwater in the study area is from southwest to northeast. The study area is characterized as high transmissivity value with an average 10,000 m^2^/day, and its hydraulic conductivity is about 100 m/day (Attia, 1985; Kotb, 1988).

## Methodology and techniques

### Potentially toxic metals (trace elements) analyses

A set of representative samples consisting of 35 drainage and 21 groundwater samples were collected, acidified in the field using concentrated HNO_3_, and analyzed. Physicochemical properties of the collected samples were determined including electrical conductivity (EC), pH, temperature, total dissolved solids (TDS), and dissolved oxygen (DO) using portable probes. In accordance with ASTM (2002), ICP (inductively coupled plasma) at Desert Research Centre (DRC) was used for potentially toxic metals (PTM) including Ag, Al, B, Ba, Cd, Co, Cr, Cu, Fe, Mn, Mo, Ni, Pb, Si, Sr, V, and Zn. All parameters are expressed in milligrams per liter.

The procedures of both quality assurance and quality control (QA/QC) were performed using standard reference material with samples duplication protocol in addition to blank samples throughout all the measurements of trace elements.

### GOD vulnerability modeling using GIS

Among the numerous vulnerability modeling methodologies, GOD was utilized in this study (Oroji, 2018; Oroji & Karimi, 2018). GOD is an empirical technique for evaluating the vulnerability of aquifers to contamination. GOD index is comprised of three parameters including aquifer type, the unsaturated zone lithology, and depth to groundwater surface. Every rating and weighting is multiplied for each factor and final vulnerability map is produced classifying the area under investigation based on degree of vulnerability as indicated in Table 1.

**Table 1:** Interval values of GOD index and corresponding classes (Murat et al., 2003)

**Class vulnerability**	Very low	Low	Average	High	Very high
**Index**	0–0.1	0.1–0.3	0.3–0.5	0.5–0.7	0.7–1

A retrieved data from literature concerning hydrogeology, geology, soil properties, and geomorphology was acquired before beginning specific data collection. This data is served as a base for organizing the field data collection (Tadesse et al., 2013). Each indicator and how to determine it are described below.

The depth (D) index indicates the distance between the surfaces of the land depth to saturated zone of the aquifer and it represents the unsaturated zone thickness that facilitates the infiltration of water to pass toward the aquifer’s saturation zone (Witczak et al., 2004). It measures the degree of contact between the contaminant percolation and subsurface materials, and consequently, the extent of physical and chemical attenuation and their degree are affected by this factor.

**Fig. 2:**
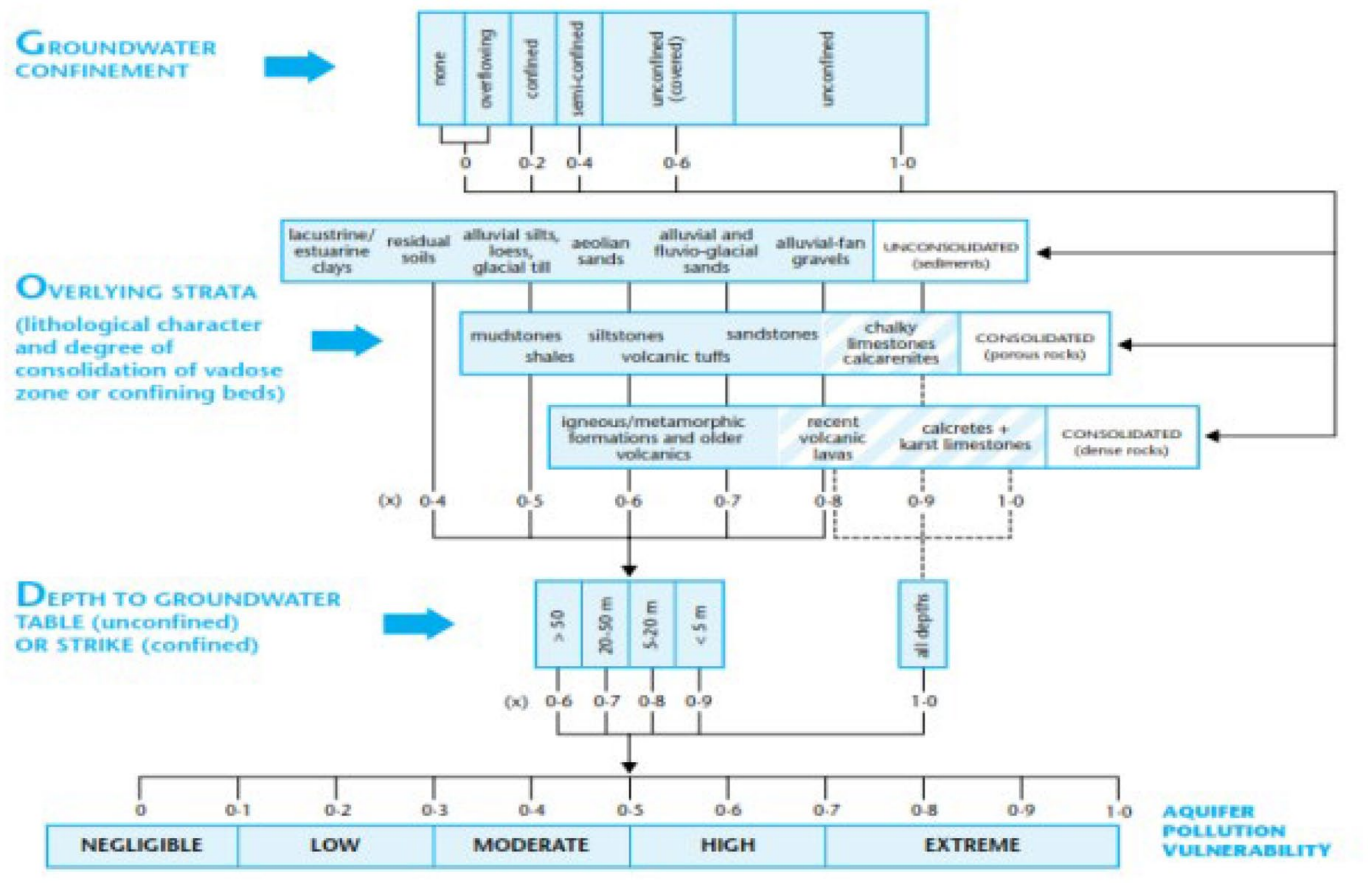
GOD method for determining the level of groundwater vulnerability (Foster, 1987)

In our case, the groundwater level measured in 21 wells in the Quaternary aquifer was subtracted from the topographic elevation in the appropriate cell site to get the depth of the groundwater distribution (D). The Kriging method was used to interpolate the depths of the groundwater. After being created, a raster map was divided into ranges according to the GOD model shown in Fig. 2. The third element is the confinement of the aquifer and the second parameter is the overall strata that describes the lithological character and the degree of consolidation of the vadose zone. For the purpose of assessing vulnerability, all of this data were imported into the GIS platform as raster maps.

### Unsaturated zone modeling

The unsaturated zone (vadose zone) was investigated in many groundwater pollution studies (Szymkiewicz et al., 2018, 2019; Selker et al., 1999; Sousa et al., 2013). This zone is acting as a filter which eliminates or attenuates (by absorption process) the pollutants such as hazardous wastes, fertilizers, and pesticides. The most common and popular software that is concerned with unsaturated zone is HYDRUS computer program (Šimůnek, et al., 2009), which has been used in this study. The equation describing vertical flow in one, two, and three dimensions which simulates water, heat, and solute transport in variably saturated porous media on the basis of the finite element method is given in (Šimůnek et al., 2009) as follows:

1 $$\frac{\partial C}{{\partial t}} = DL\frac{{\partial^{2} C}}{{\partial x^{2} }} - vx\frac{\partial C}{{\partial t}} - \frac{\rho }{\theta }\frac{\partial C}{{\partial t}} \pm \left[\kern-0.15em\left[ {\frac{{\partial C^{*} }}{\partial t}} \right]\kern-0.15em\right]rxn$$

where *C* (mg/L) is the concentration of solute in liquid phase, *t* (h) is the time, *DL* (cm^2^/h) is the longitudinal dispersion coefficient, *vx* (cm/h) is the average linear groundwater velocity, *ρ* (g/cm^3^) is the bulk density of aquifer, *θ* is the volumetric moisture content or porosity for saturated media, C* (mg/g) is the amount of solute sorbed per unit weight of solid, and *rxn* is the subscript indicating a biological or chemical reaction of the solute (other than sorption).

It also includes information about the concentration of solute in liquid phase, longitudinal dispersion coefficient, the average linear groundwater velocity, the bulk density of aquifer, and volumetric moisture content or porosity for saturated media. This model also accounts for the biological or chemical reaction of the solute (other than sorption).

## Results and discussion

### PTMs and their distribution in the study area

The main sources of the PTM include agricultural and industrial wastewater effluents, fertilizers, mining effluents, and fossil fuels (Fostner & Wittman, 1981). Unfortunately, although they exist in nature in low concentration, low amounts of trace elements above permissible limits can lead to harmful potential risks for human health as they have particularly low decaying nature and accumulate in food chains. Although they are strongly attached and bound to the soil particles, they are moving down to groundwater by leaching. Hence, their natural concentration in fresh water varies accordingly. Few investigations revealed a link between the concentration of trace elements in water and in various cancer types. There is a growing goal for the reuse of treated sewage water in land reclamations in Egypt and many other countries. Even if the nutrients in sewage water are promising source for fertilizer, its contamination is thought to be one of the major problems associated with the irrigation with sewage. Since heavy metals are strongly bound by the soil, leaching of metals downward into groundwater may cause serious hazards. The statistical summary of trace element values for drainage and groundwater samples in comparison to the international standards for drinking purposes (WHO, 2017) is given Table 2.

**Table 2:** Statistical summary of trace element results for collected water samples

	**Element**	**Min**	**Max**	**Average**	**WHO (**2017**)**		**Min**	**Max**	**Average**
**Groundwater**	**Ag (mg/l)**	0.006	0.054	0.030		**Drainage water**	0.009	0.093	0.033
	**Al (mg/l)**	0.023	0.776	0.231	0.20		0.028	5.703	0.654
	**B (mg/l)**	0.008	0.192	0.072	1		0.030	0.189	0.084
	**Ba (mg/l)**	0.019	0.286	0.139	2		0.003	0.165	0.074
	**Cd (mg/l)**	0.003	0.026	0.010	0.003		0.001	0.040	0.012
	**Co (mg/l)**	0.002	0.041	0.016	< 0.05		0.002	0.036	0.015
	**Cr (mg/l)**	0.027	0.051	0.041	0.05		0.011	0.043	0.025
	**Cu (mg/l)**	0.008	0.216	0.084	1		0.014	0.202	0.070
	**Fe (mg/l)**	0.035	1.762	0.609	0.3		0.020	5.622	1.099
	**Mn (mg/l)**	0.019	2.273	0.601	< 0.1		0.003	0.376	0.222
	**Mo (mg/l)**	0.004	0.106	0.055			-0.044	0.117	0.055
	**Ni (mg/l)**	0.002	0.056	0.033	0.01		0.005	0.059	0.030
	**Pb (mg/l)**	0.041	0.160	0.117	0.01		0.009	0.266	0.094
	**Si (mg/l)**	5.253	20.390	11.046	0.01		0.151	9.789	5.889
	**Sr (mg/l)**	0.110	1.748	0.710	0.11		0.023	8.370	1.176
	**V (mg/l)**	0.018	0.117	0.056			0.012	0.203	0.059
	**Zn (mg/l)**	0.002	0.570	0.151	3.00		-0.005	0.524	0.094

**Fig. 3:**
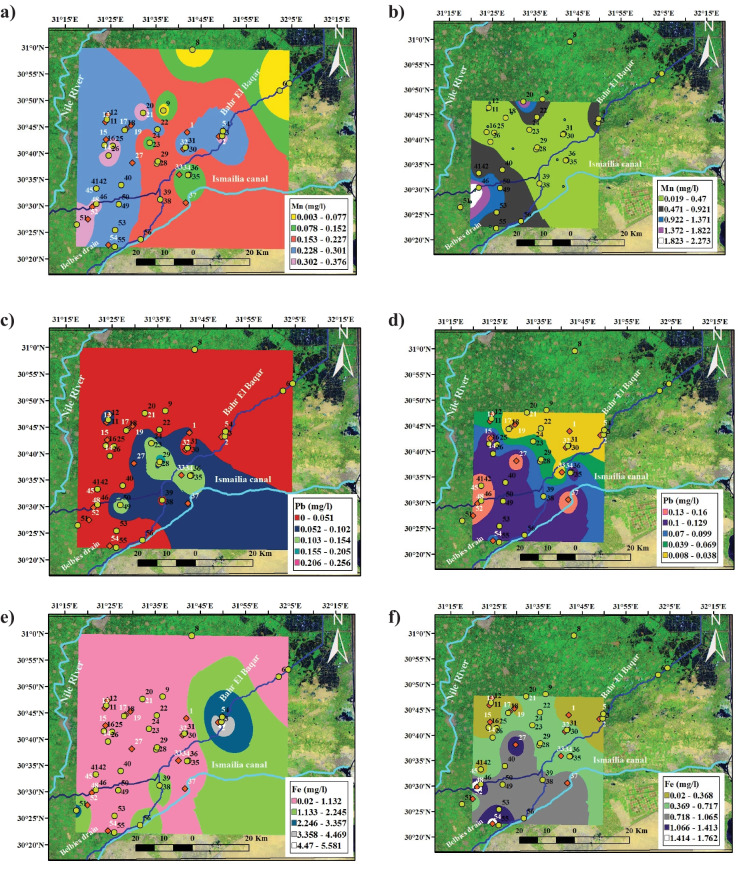
Spatial distribution of Mn, Pb, and Fe in drainage **a**, **c**, and **e **and groundwater samples **b**, **d**, and **f**

It was observed that some trace elements exceeded WHO limits for drinking purposes including Fe, Mn, and Pb for drainage and groundwater samples as follows:

- The concentrations of iron in the analyzed groundwater and drainage water samples vary from 0.03–1.76 and from 0.02–5.62 mg/l, respectively. All these samples exceed the maximum recommended limit for Fe^3+^ in drinking water (0.3 mg/l). According to WHO (1984), excess amount of iron is stored primarily in the liver and bone marrow resulting in many dangerous diseases. The main contamination sources of iron results from using them used as coagulating agents in water treatment plants where cast iron, steel, and galvanized iron pipes are used for water distribution.
- The concentrations of Mn^2+^ in drainage and groundwater vary from 0.002 to 0.376 and 0.018 to 2.27 mg/l, respectively. Most of these samples exceed the maximum recommended limit for Mn^2+^ in drinking water. This may be due to discharge of industrial effluents from the located steel and iron factories, sewage, and acid-mine drainage.
- Lead concentrations varied between 0.04 and 0.159 mg/l and 0.009 and 0.266 mg/l in drainage water and groundwater, respectively, which is exceeding the permissible limit of lead in drinking water (0.01 mg/l) (WHO, 2017). Lead is considered one of the first non-ferrous metals used by humanity. It is used in many industrial applications such as batteries and cable sheeting. Lead does not appear to be an essential element for life for any organism. It is less toxic to plants than mercury and copper, with adverse effects being noted at concentration between 100 to 5000 µg/l. Its toxicity is rising from its substitution of calcium in human bone and accumulates in it. Lead poisoning can lead to anemia, kidney disease, and disturbances of the central nervous system and can cause mental retardation and convulsions in later life of children (Fig. 3).

### Groundwater vulnerability assessment

**Fig. 4:**
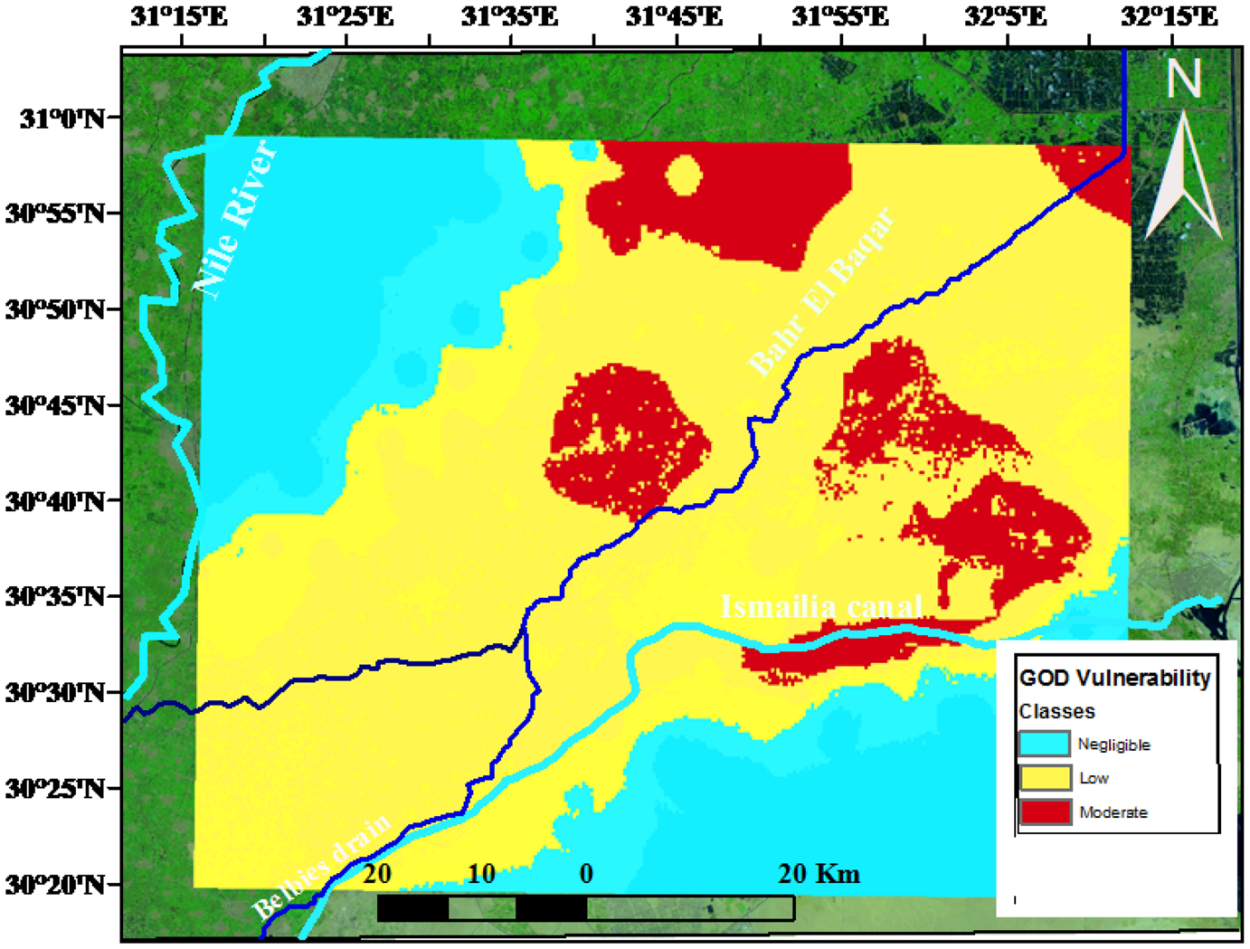
Vulnerability map classes in the study area.

The vulnerability maps were created after mapping every indicator by superimposing the individual maps and computing the indices on a grid map. According to the equation, the vulnerability index for each grid cell was determined as the weighted total of the indicators. The hydrological settings shown on the map must be evaluated and the areas on the final map are then given the proper hydrogeological setting labels. Vulnerability indices of all models are calculated and the final vulnerability map is separated into groups according to vulnerability levels (Fig. 4).

The most vulnerable area was recorded between the low vulnerability and moderate vulnerability classes at the central parts of the governorate while northwestern part and southeastern parts are negligible vulnerability class. The verification of the vulnerability maps was done using the spatial distribution maps of the Pb, Fe, and Mn as shown in Fig. 3. A good match was observed between the vulnerable areas and the highest concentration of trace elements distribution revealing that the main source of these contaminations is anthropogenic effects and industrial wastes.

### Contaminants transport in unsaturated zone modeling

The protection of unsaturated zone is essential for security of groundwater quality especially for shallow aquifers in arid areas. So, demarcation of pollutant changes using models in this zone can enhance the accuracy of groundwater quality assessments. Potentially toxic metals (PTMs) concentration in groundwater is a key factor for health risk assessment. In this study, a degree of contamination for Fe, Pb, and Mn was found as in some surface and groundwater samples in the study area. Those elements were simulated under dynamic vadose zone conditions using HYDRUS model for better understanding of their behaviors. Such modeling is useful for the characterization of trace elements evolution and for implementing vadose zone and groundwater remedial activities.

**Fig. 5:**
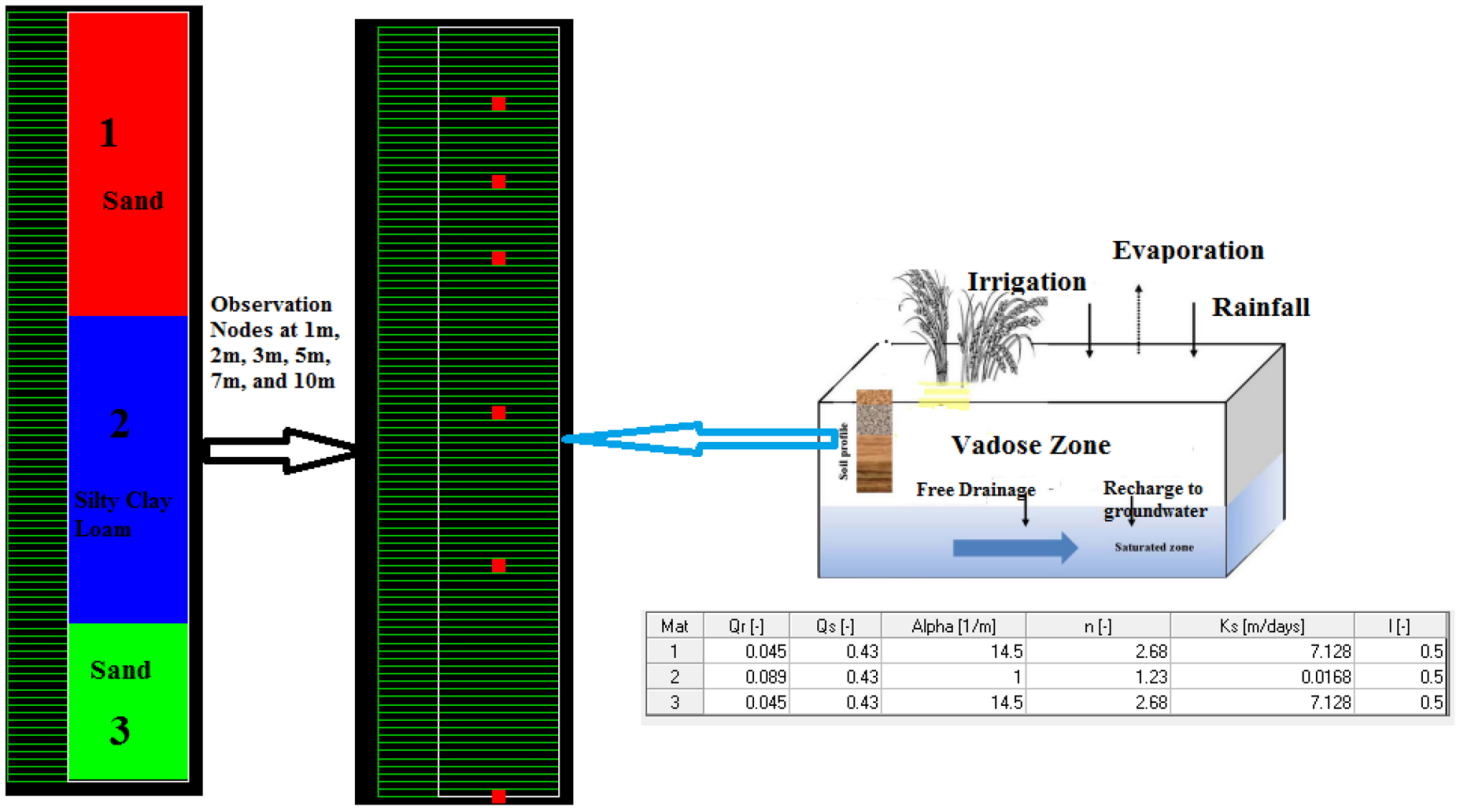
Layered soil profile with some conceptualization illustrations

Vertical water flow from wastewater via irrigation and solute leaching were simulated using HYDRUS-1D below unsaturated zone thickness of 10 m. Fe, Pb, and Mn have been used as contamination sources at the top of the unsaturated zone for 10 years simulation time. Precipitation and evapotranspiration were inserted in the model as input for time variable boundary conditions. The total depth of soil profile was estimated to be 10 m and length units are illustrated in Fig. 5. Soil profile was subdivided into 0–4 m, 4–8 m, and 8–10 m through three sections based on literature work of hydrogeology survey in the study area (El Sayed et al., 2011). Precipitation and potential evapotranspiration fluxes were assumed to be an atmospheric boundary at the top boundary, whereas free drainage conditions that may be attributed to the groundwater table were considered for the bottom boundary. Concentration flux boundary for the transport of the three elements was used as the upper and lower boundary conditions under the existence of initial soil water as initial conditions for leaching simulation for conservative consideration. All hydraulic properties were initially estimated by default properties in the HYDRUS model.

**Fig. 6:**
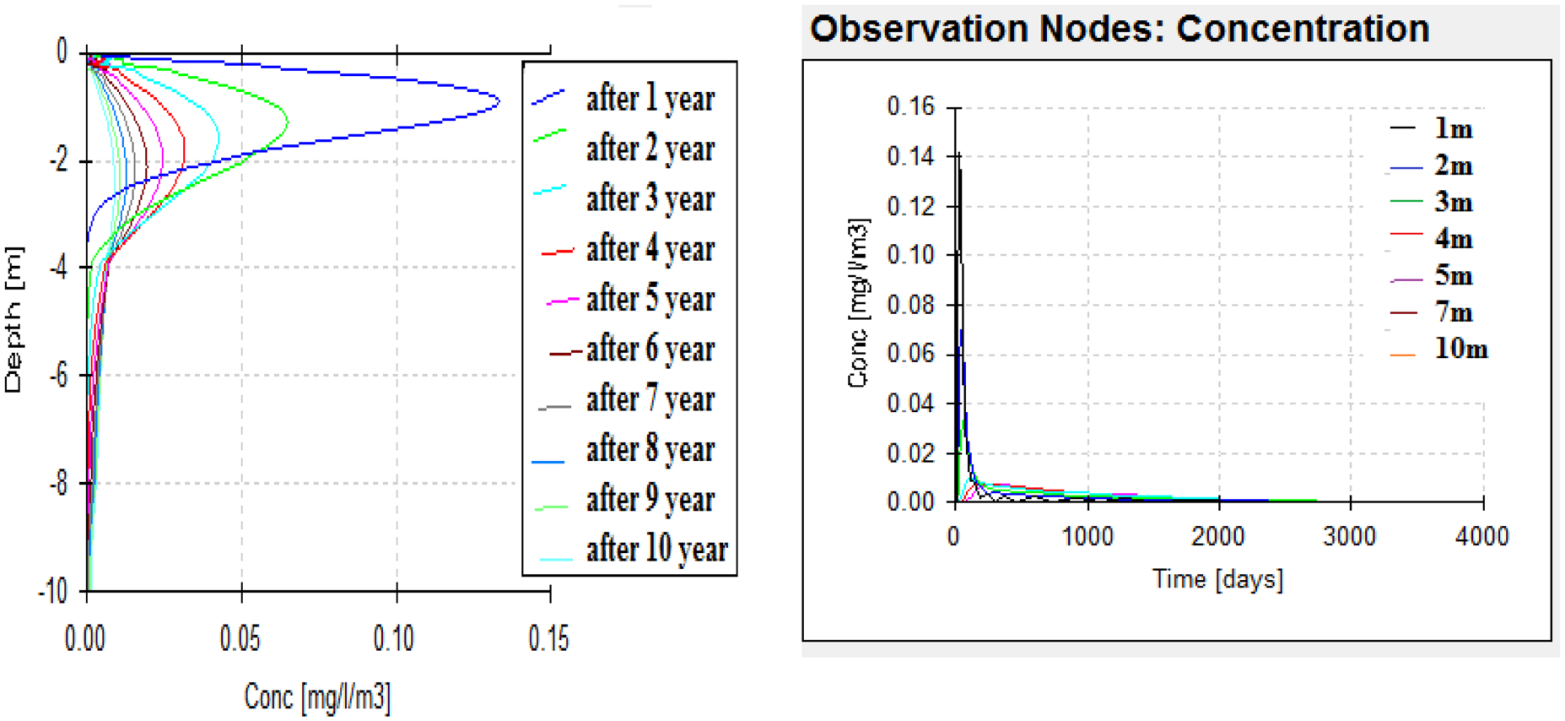
Fe transport in unsaturated zone at different nodes (depths) (1, 2, 3, 5, 7, and 10 m) and times

**Fig. 7:**
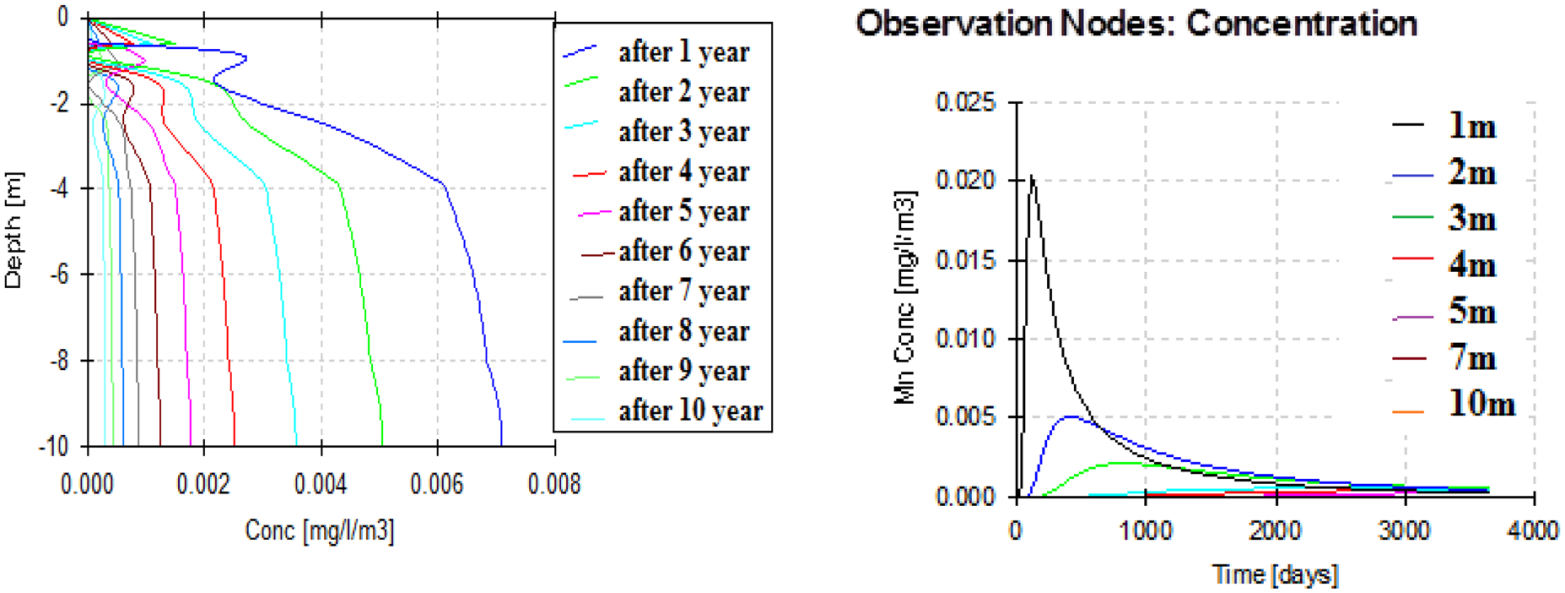
Pb transport in unsaturated zone at different depths (1, 2, 3, 5, 7, and 10 m) and times

**Fig. 8:**
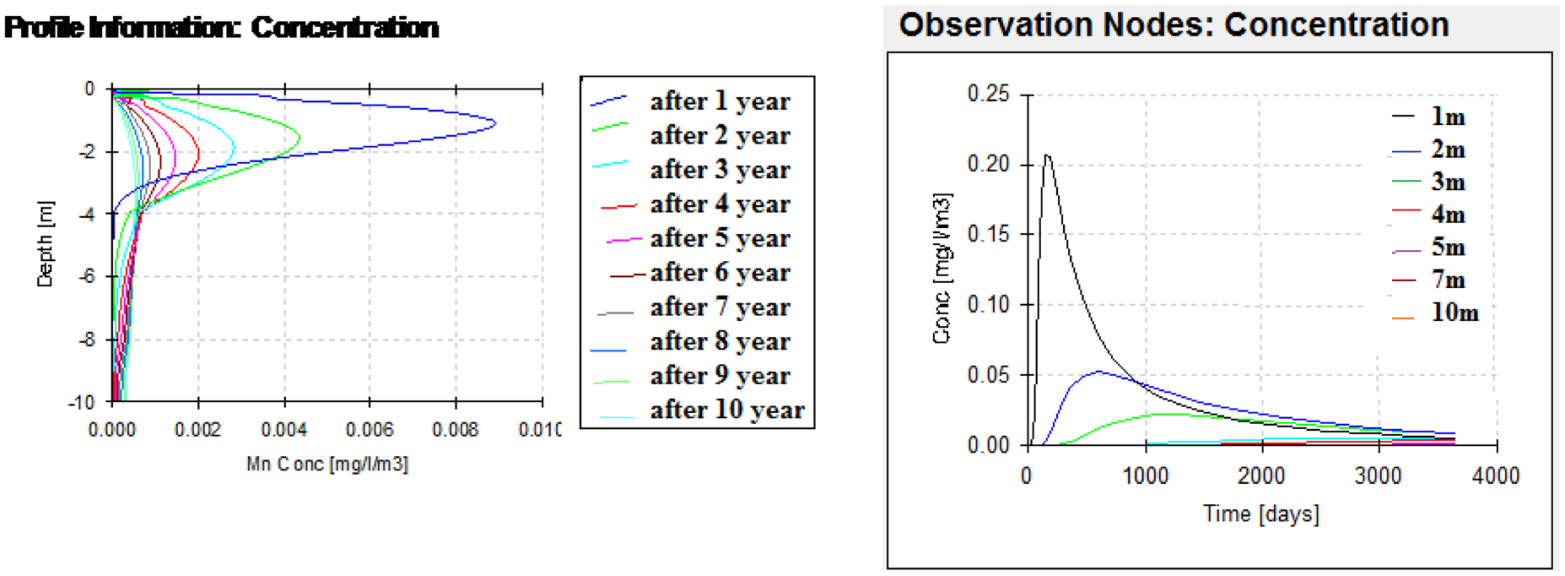
Mn transport in unsaturated zone at different depths (1, 2, 3, 5, 7, and 10 m) and times

After HYDRUS model conceptualization, the case was simulated to obtain the outputs at several nodes (1 m, 2 m, 3 m, 5 m, 7 m, 10 m). From the depth profile (Figs. 6, 7, and 8), it can be noticed that the maximum peak concentrations of Fe, Pb, and Mn are decreasing through the depth profile to reach negligible concentration at the end of the unsaturated zone depth. This has occurred under different initial concentrations and different behavior of these elements in the soil profile matrices according to their different K_d_ (partitioning coefficients) of each element. The retardation in soil is higher in the case of Pb than Fe which has the max peak concentration at the end of the simulation. On the other hand, according to the time profile concentration (Figs. 6, 7, and 8) of Fe, Pb, and Mn, it is maximum at nodes in the sand profile and delayed behavior is noticed at nodes in the silty clay loam profile (Fig. 6). The migration of solutes downward in fine texture soils is usually slower compared to coarse textured one as a result of lowering hydraulic conductivity in finer ones. The concentration of Fe, Pb, and Mn reaches to 0.00158 mg/l, to 0.00031 mg/l, and 0.0089 mg/l, respectively, at the end of the simulation (after 3650 days) in the unsaturated zone at the lower boundary.

## Conclusion and recommendations

The vulnerability map that was obtained during this work determined the sectors that are severely threatened by leakage from wastes through the percolation of contaminants. The study area was classified into three categories. Negligible vulnerability was observed at southeastern and northwestern parts. On the other hand, the rest of the study area was mostly ranged between low vulnerable areas while some sporadic areas in the central part were classified as intermediate vulnerability class. The deduced map was then verified by comparing it with the spatial distribution of some trace elements in the study area. The contaminants transport in unsaturated zone was modeled using HYDRUS model for selected trace elements that exceeds the permissible limits for drinkable water (Pb, Fe, and Mn), in a trail to demarcate the pollution extent of these elements in vadose zone. This can help in mitigation action for groundwater conservation from pollution. It was found that those elements penetrate the unsaturated zone completely and further pollution is expected to occur. These findings are critical for integrated water resource management in the study area, while there is still more action needed to be implemented to protect the environment.

Some recommendations are given below for maintaining the health to reduce water pollution from these sites.

- Adapting proper management measures that include adequate wastewater treatment from industries.
- Continuing to monitor lithological data through the construction of more suitably spaced network of monitored wells based on more vulnerable areas in the study area to determine the most likely pathway of contaminants.
- Controlling and restricting pesticides and agricultural fertilizers usage by farmers and land owners through setting some governmental restrictions.
- Lining waste drains and surface canals to protect the surrounding groundwater system.
- Reducing overpumping for the wells to avoid salinization problems with respect to the distance between wells.

## Data Availability

All data generated or analyzed during this study are included in this manuscript. All authors have read, understood, and have complied as applicable with the statement on “Ethical responsibilities of Authors” as found in the Instructions for Authors and are aware that with minor exceptions, no changes can be made to authorship once the paper is submitted.
